# The Swedish national guideline on immediate and uninterrupted skin-to-skin contact and mother-newborn couplet care

**DOI:** 10.3389/fped.2026.1937937

**Published:** 2026-09-10

**Authors:** S. Klemming, Vesta Seyed Alikhani, K. Breding, P. Bäcke, S. Elvermark, A. Gustafsson, Å. Hermansson, E. Jonsson, N. Kjellqvist, M. Mattisson, M. Nelander, M-C. Nilsson, A. Nyholm, S. Lilliesköld, Y. Thernström Blomqvist, E. Wilson, S. Arwehed

**Affiliations:** 1 NIDCAP Trainer with the NIDCAP Federation International, Stockholm, Sweden; 2 Department of Neonatology, Sahlgrenska University Hospital, Gothenburg, Sweden; 3 Department of Obstetrics, Sahlgrenska University Hospital, Gothenburg, Sweden; 4 Department of Women’s and Children’s Health, Uppsala University, Uppsala, Sweden; 5 Department of Neonatology, Uppsala University Children’s Hospital, Uppsala, Sweden; 6 Department of Anesthesiology, Division of Obstetric and Gynecologic Anesthesia and Intensive Care, Danderyd Hospital, Stockholm, Sweden; 7 Department of Women’s and Children’s Health, Karolinska Institutet, Stockholm, Sweden; 8 Department of Obstetrics and Gynecology, Linköping University Hospital, Linköping, Sweden; 9 Department of Women’s Health, Karolinska University Hospital, Stockholm, Sweden; 10 Department of Anesthesiology and Intensive Care, Kalmar Hospital, Kalmar, Sweden; 11 Department of Neonatology, Växjö Hospital, Växjö, Sweden; 12 Department of Obstetrics, Uppsala University Hospital, Uppsala, Sweden; 13 Department of Obsterics and Gynecology, Ystad Hospital, Ystad, Sweden; 14 Department of Ambulance Services, Region Västerbotten, Umeå, Sweden; 15 Department of Neonatology, Astrid Lindgren’s Children’s Hospital, Karolinska University Hospital, Stockholm, Sweden

**Keywords:** infant health, innovative therapies, maternal health, maternal-newborn care, mother-newborn couplet care, neonatal care, non-pharmacological interventions, preterm and low birth-weight infants

## Abstract

Immediate and continuous skin-to-skin contact between a mother and her newborn is associated with numerous health benefits. Benefits for infants includes improved thermoregulation, stabilization of heart rate and respiration. Benefits for mothers include reduced maternal postpartum bleeding and improved maternal blood pressure regulation. Benefits for both include reduced stress, earlier initiation of breastfeeding, and enhanced mother–infant bonding. There is a need to scale-up the clinical implementation of skin-to-skin contact for all newborns across care settings. In particular, current practices should be reconsidered for infants born preterm, with low birth-weight, requiring specialized medical interventions, and after instrumental births/caesarean sections. This also requires reorganization of maternal and newborn care. As emphasized by the World Health Organization in its implementation strategy for scaling up kangaroo mother care (2023), the mother-newborn dyad should be regarded as an *inseparable center* around which health care is organized, enabling zero separation. This article presents a translation of a Swedish national guideline recommending immediate and uninterrupted skin-to-skin contact for at least the first 2 hours after birth for all newborns, followed by a mother–newborn couplet care model including continued skin-to-skin contact. In mother-newborn couplet care the mother and her newborn, regardless of medical needs or clinical complexity, are cared for together in the same physical environment to minimize separation. Implementation requires multidisciplinary collaboration across operations, clearly defined responsibilities, and access to appropriately adapted facilities, equipment, and care processes across all settings in which mother-newborn dyads receive care. The guideline is intended for healthcare professionals and managers across the continuum of maternal, obstetric, neonatal, anesthetic, surgical, and intensive care in Sweden. It applies to all mothers and newborns during the first hours after birth and throughout the subsequent hospital care when both are managed in a hospital setting. The guideline includes a scientific rationale, definitions, recommendations, implementation guidance, impact assessment and quality indicators for monitoring and evaluation. The original Swedish version of this guideline was published in 2024. It has been reported to be helpful in the implementation in Swedish hospitals. We recommend others to make similar national, regional and/or local guidelines to support implementation of these innovative, non-pharmacological interventions to improve maternal and infant health.

## 1 Introduction and background to the English translation

This is a translation and reprint of the national guideline “Initial obruten hud-mot-hudkontakt och samvård” originally published online September 2024 in the Swedish language on the clinical knowledge support platform for Swedish healthcare: 1177 Vårdguiden/vårdpersonal.

The original guideline was written by the author group under the Swedish Association of Local Authorities and Regions and the National Program Area for Children’s and Adolescent’s Health. Stina Klemming prepared this translation and adjustment for publication and the co-authors from the original author group reviewed the English version. Permission to reprint this English translation was granted by the Swedish Association of Local Authorities and Regions and the National Program Area for Children’s and Adolescent’s Health.

Swedish maternal and newborn care follow high standards and quality. For 30 years there has been a strong development towards humanization of care and an infant- and family-centered developmental approach. In 2022 when the World Health Organization updated the recommendations for preterm and low birth-weight infants, separations between newborns and mothers were still common in Sweden during the first hours after birth. This was especially true for newborns born preterm, with low birth-weight or in need of specialized medical interventions, and/or after instrumental births/caesarean sections as well as for mothers with peripartum complications. To promote a re-think and new practices on skin-to-skin contact and zero separation between mothers and newborns and to promote equitable and evidence-based maternal-newborn care, the National Program Area for Child and Adolescent Health initiated a project for a national guideline through the National system for knowledge-driven management within Swedish healthcare. The project was governed by the Swedish Association of Local Authorities and Regions under the National Program Area for Children’s and Adolescent’s Health and was supported by the National Program Area for Gynecological Diseases, Pregnancy and Childbirth, and the National Program Area for Anesthesiology, Intensive Care, and Transplant medicine. The guideline was developed through a collaborative process with an author group of healthcare professionals from neonatal care, obstetrics, anesthesiology, and intensive care, and one patient representative. An extensive prepublication review was conducted through invitations to all Swedish healthcare regions and specialty societies across the care continuum, including maternal, obstetric, postpartum and postnatal, neonatal, anesthetic, surgical, and intensive care. Input was also obtained from several patient and parent organizations. The guideline was launched through an open-access webinar presenting its content and facilitating discussion on shared future directions for maternal and newborn health, including reflections on key challenges and opportunities. The original guideline published in Swedish consists of three documents; 1. The Swedish guideline for immediate and uninterrupted skin-to-skin contact and mother-newborn couplet care; 2. A scientific background; and 3. An impact assessment. In this translated version the three documents are adjusted to one.

## 2 Scope of the recommendations

This guideline addresses staff and managers in the care chain of maternal health care, obstetric care, postpartum- and postnatal care, neonatal care, anesthesia, surgery and intensive care. The recommendations apply to all newborns and their mothers during the first hours after birth and the following time when both are being cared for in a hospital setting.

The guideline intends to provide guidance for the implementation of immediate and uninterrupted skin-to-skin contact and mother-newborn couplet care. The goal is that new mothers and their newborns are cared for together regardless of medical needs and complexity and with early or immediate initiation of skin-to-skin contact. Based on current scientific knowledge, the recommendation for immediate skin-to-skin contact directly at birth applies to newborns from 28 + 0 weeks of pregnancy. To achieve the goals of the recommendations in this guideline, consensus and cooperation across operational boundaries, a clear division of responsibilities and appropriate and adapted premises, equipment and working methods are required. The guideline includes definitions, recommendations, and guidance for the implementation and development of working methods around immediate and uninterrupted skin-to-skin contact and mother-newborn couplet care.

The recommendations were made within the framework of the National system for knowledge-driven management within Swedish healthcare and are based on other Swedish and international guidelines and scientific evidence (1–8). The recommendations are also based on existing routines in Swedish hospitals and on experiences in the multiprofessional author group. The group have representation from all healthcare regions in Sweden, from all specialty associations within the care chain, maternal healthcare, obstetric care, postpartum- and postnatal care, neonatal care and anesthesiology, surgery and intensive care, as well as from a patient representative.

In this guideline the word “mother” has been chosen to refer to the birthing parent and the person who gave birth, although not everyone identifies as a woman or a mother (9). Families come in many constellations and parenting can take many forms. In the care situation, inclusive language needs to be used based on the person’s expressions and wishes. For more information, see Recommendation: “Inclusive language” from The Swedish Federation for Lesbian, Gay, Bisexual, Transgender, Queer and Intersex Rights (10).

## 3 Scientific background

The first hours and days after birth are unique and are defined as a sensitive and critical period for both mothers and newborns based on the mutual regulation of physiological, behavioral and psychological processes. All newborns and new parents have a need for - and a right to - physical and emotional closeness (8, 11–13) and separation should be avoided (14–16). Mother-newborn couplet care has the potential for positive health effects (17–31). In the Swedish National Board of Health and Welfare’s guidelines for newborn care and for pregnancy, birth and the time after birth it is particularly emphasized that healthcare facilities should ensure that newborns and parents are not separated at or after birth and that immediate and uninterrupted skin-to-skin contact between a mother and a newborn is enabled to the greatest extent possible (1–3).

Immediate and uninterrupted skin-to-skin contact between a mother and her newborn right at birth has many scientifically proven positive effects (5, 16, 32–36). This applies regardless of birth mode (33, 37). For newborn infants, skin-to-skin contact has been shown to stimulate newborn instinctive behaviors and the initiation of breastfeeding (32, 38–41), stabilize temperature regulation (32, 42), blood sugar regulation (43), and cardiorespiratory regulation during the newborn’s physiological adaptation after birth (44, 45). Likewise, beneficial effects on the microbiota, the normal bacterial flora (46), eating behaviors (33) sleep and self-regulation (47) are described. For the mother, skin-to-skin contact has been shown to stimulate lactation (38) and uterine contraction for less postpartum bleeding (48–51), stabilize blood pressure regulation (52), and strengthen emotional attachment to the newborn (53, 54). Skin-to-skin contact and couplet care from birth have been shown to improve experiences for both mothers and newborns (21, 55) with positive effects on well-being (56, 57), reduced stress (58), anxiety and pain (22, 23, 48, 56, 59–62). Skin-to-skin contact has also been shown to reduce stress and anxiety for the other parent (21, 55, 60). A positive development for a confident parenting is seen by keeping the family together (24, 63, 64).

Immediate and uninterrupted skin-to-skin contact and mother-newborn couplet care describe the place of care. The content of the care is guided by the clinical condition of the mother and the newborn and should align with existing guidelines and clinical routines in maternal and newborn care. Immediate and uninterrupted skin-to-skin contact can be implemented within current recommendations for umbilical cord clamping in infants delivered after 34 weeks of gestation who do not require resuscitation (65). This approach also applies to the stabilization of transition at birth for infants born before 35 weeks of gestation (66). Evidence indicates that routine procedures, such as weighing and measuring the infant, can be deferred until after the first hours following birth (4). During this period, the maternal care can be adapted to support immediate and continuous skin-to-skin contact (17).

In this guideline, uninterrupted skin-to-skin contact for at least 2 hours is recommended. This is to allow time for the newborn’s instinctive behaviors to take place (40, 41, 67, 68) At the same time, consideration must be given to the importance of the mother emptying her bladder. The newborn’s physiological adaptation is promoted by uninterrupted skin-to-skin contact with a parent during the first six hours (42, 44, 69, 70). Continued skin-to-skin contact during the first hours, days and throughout the hospital stay and beyond has strong scientific support and should be encouraged and supported as much as possible.

In cases where interruptions in skin-to-skin contact between a mother and her newborn is needed during the first hours, studies have shown positive effects of the newborn being cared for in skin-to-skin contact with the other parent, or a person appointed by the parent (42, 44, 63). If immediate and uninterrupted skin-to-skin contact with the mother is not possible until the newborn breastfed, breast stimulation, hand expression and colostrum administration should be encouraged as soon as possible, preferably already during the first hour (71, 72). When skin-to-skin contact between the mother and the newborn resumes, the newborn instinctive behaviors are stimulated again (67, 73, 74). It is also known that both full-term and preterm neonates exhibit increased breastfeeding-specific behaviors during skin-to-skin contact even after the first hours (75).

Mother-newborn couplet care together with immediate or very early and continuous skin-to-skin contact for low birth-weight and preterm newborns has reduced the risk of serious infections in low- and middle-income countries (76), lowered infant mortality (77), and resulted in better growth (78). For low birth-weight and preterm infants, intermittent skin-to-skin contact during hospitalization has shown positive and lasting effects on brain development and organization, and consequently positive effects on behavior and long-term development (79–83).

According to the World Health Organization (WHO), skin-to-skin contact should be initiated as soon as possible after birth. For low birth-weight and preterm newborns, skin-to-skin contact is recommended immediately and as continuously as possible during the hospital stay and beyond. The recommendations on initiation of skin-to-skin contact directly at birth apply to newborns from 28 + 0 weeks of pregnancy and with a birth weight over 1,000 grams (5, 33–36, 84). For the group of critically ill newborns and newborns born extremely preterm before week 28, there is a growing number of publications supporting early skin-to-skin contact and closeness between newborns and their parents (61, 85–90).

The strength and significance of these outcomes have been studied for different groups of newborns and families and differ based on starting points such as the newborn’s age, maturity and clinical condition, the mother’s condition and the family’s wishes and needs. Many of the publications we refer to in this scientific background for the guideline “immediate and uninterrupted skin-to-skin contact and mother-newborn couplet care” are systematic reviews with grading of the strength of the evidence. There is strong scientific support for immediate and continuous skin-to-skin contact and minimized separation between mothers and newborns after birth. Close physical contact between newborns and parents, and early positive experiences are important for development and health in the short and long term. The significance for the individual parent-newborn dyad cannot be assessed based on current scientific evidence.

## 4 Definitions

### 4.1 Immediate and uninterrupted skin-to-skin contact

Immediate and uninterrupted skin-to-skin contact means that the newborn lies naked directly against the mother’s bare skin for at least the first two hours after birth in order for the newborn to be undisturbed in its instinctive breastfeeding behaviors. This guideline focuses on the first hours after birth and skin-to-skin contact is recommended to continue for as many hours per day as possible during the hospitalization. When skin-to-skin contact with the mother is not possible, the other parent, or a person appointed by the parent, can care for the newborn in skin-to-skin contact.

### 4.2 Mother-newborn couplet care

Couplet care means that two patients receive their care together. With mother-newborn couplet care, the new mother and the newborn receive their care in the same unit and in close physical proximity to each other. This also applies when the mother, the newborn or both need specialized care. When either the mother or the newborn is discharged, care is no longer defined as couplet care.

## 5 Patient participation, person-centered care

Healthcare professionals have a responsibility to plan for and facilitate immediate and uninterrupted skin-to-skin contact and couplet care together with the family. Communication and information are based on individual conditions and needs. Dialogue with parents about previous experiences, needs, expectations, physical and psychological resources and social networks is a prerequisite for giving the family individual support and the opportunity for participation and informed consent. Personal privacy must be considered, which is especially important when patients are physically exposed.

Care of the mother and the newborn together in immediate and uninterrupted skin-to-skin contact and couplet care can be seen as part of a person-centered approach. Within neonatal care, this is seen as part of the concept of infant- and family-centered developmental care.

## 6 Support and communication

All expectant parents should be informed already during pregnancy about the health benefits of immediate and uninterrupted skin-to-skin contact and couplet care. Parents should be informed about the newborn’s need of closeness to a parent, or a person appointed by the parent, regardless of the condition of the mother or the newborn after birth.

Language and communication difficulties are handled individually, for example with the use of an interpreter throughout the course of care. Verbal information should be supplemented with visual and/or written material in different languages. Information should be repeated and communicated via maternal health care, obstetric care, postpartum- and postnatal care, neonatal care, parent groups, national and regional healthcare websites.

Communication and support to the new parents continues to be an important focus for healthcare professionals throughout the hospital stay. If families are separated, digital solutions facilitate communication within the family and between parents and staff.

## 7 Requirements

### 7.1 Goal statement

The goal of all personnel involved in care before, during and after birth should be that all new mothers and their newborns are cared for together, regardless of medical needs and complexity.

### 7.2 Organization

Each healthcare management is responsible for ensuring that all operations and departments that are part of the care chain receive a clear and feasible mission through regional and local prioritization. The operations and departments are individually and collectively responsible for enabling immediate and uninterrupted skin-to-skin contact and couplet care for all mothers and their newborns.

Important organizational components at national, regional and local level are:

- shared overall vision and goals
- collaboration between the management groups for different operations
- clear division of responsibilities between operations and disciplines
- guaranteed resources, staffing and competence at all hours
- common, consistent guidelines and routines for coordination of care
- systematic follow-up based on indicators.

### 7.3 Skills, training and communication

Immediate and uninterrupted skin-to-skin contact and couplet care require flexible care teams across operational boundaries to enable care where the patient is, as well as well-informed parents who expect to be with their newborn.

This is achieved by:

- well-functioning information and communication channels, including alarm routines within and between different units
- responsibility for care of each patient in each situation is made clear to everyone
- staff at the different units can delegate and be delegated certain tasks according to locally developed guidelines
- regular multiprofessional simulation exercises with exchange of experience across operational boundaries, wards and disciplines
- auscultation visits by staff between the units are used to increase motivation, responsiveness, consensus and flexibility
- parents are well prepared, see section “Support and communication”.

### 7.4 Premises and equipment

Premises need to be designed for both routine care and emergency situations and have established alarm systems for each patient. Wards and rooms of all kinds should be adapted to offer a good environment for newborns, parents and staff. This includes, among other things, ergonomics, conditions for good care hygiene and personal integrity for the mother and the newborn. Conditions for immediate and uninterrupted skin-to-skin contact and couplet care should be included in the planning of renovations and constructions in obstetrics, neonatology, anesthesia, surgery and intensive care.

Equipment for safe and adequate care of both mothers and newborns, both during routine care and in emergency situations, needs to be available in or adjacent to the birth room and in all care units where mothers and newborns can be cared for in couplet care.

Equipment for both the mother and the newborn should be mobile and include, among other things:

- medical equipment for initial stabilization, emergency situations and continued care
- materials, aids and medicines for care, treatment and nursing
- monitoring equipment for physiological parameters
- the possibility of transferring the mother and the newborn within the hospital while maintaining skin-to-skin contact.

## 8 Level of care and collaboration

Before, during and after birth, the following questions should be answered by the team:

- How can immediate and uninterrupted skin-to-skin contact for the mother and the newborn be made possible?
- If there is a justified interruption in skin-to-skin contact with the mother, can the newborn be cared for in skin-to-skin contact with the other parent or with a person appointed by the parent?
- If a place of care other than skin-to-skin is deemed necessary, when and how will a new position on care in skin-to-skin contact be taken?
- How and where can the mother and the newborn be cared for together?
- If couplet care is not possible initially, what is the obstacle and when can the situation be reassessed?

### 8.1 Assessment of the level of care

Most mothers and newborns are healthy and adaptations can be made without much difficulty to provide access to immediate and uninterrupted skin-to-skin contact and couplet care after birth. Individual assessments of the level of care and place of care are based on the medical and nursing needs of the mother and the newborn.

Couplet care is planned on the unit responsible for the patient with the most complex medical needs. Care is tailored based on each patient’s medical condition together with the unit’s capacity and conditions. The assessment is repeated if a patient’s condition, or the capacity and conditions of the unit changes. If separation needs to occur, couplet care and skin-to-skin contact should be resumed as soon as possible. Parents should be continuously involved and informed about the current assessment.

To assess the level of care, tools such as postpartum/postnatal risk assessment of both the mother and the newborn can help individualize care.

## 9 Implementation

### 9.1 Immediate and uninterrupted skin-to-skin contact

Skin-to-skin contact should be initiated as soon as possible after birth. To optimize the physiological transition after birth and support breastfeeding initiation, initial skin-to-skin contact with the mother should be prioritized. If this is not possible, skin-to-skin contact should be initiated with the other parent, or with a person designated by the parent. In multiple births, both newborns are placed together with the mother if possible. Routine care of the mother and the newborn should, if possible, be postponed until after the first two hours and medically justified care, nursing and transfers should take place in uninterrupted skin-to-skin contact.

To provide immediate and uninterrupted skin-to-skin contact regardless of mode of birth, follow the steps below:

1. Prepare the parents and inform about the importance of and the practicalities of immediate and uninterrupted skin-to-skin contact. Also be sensitive to the parent’s individual needs.
2. Prepare with adequate equipment and materials for care and nursing at the place of birth. Adjust the room temperature and ensure access to warm textiles. In the case of a caesarean section - if possible, place iv-lines, sensors for oxygenation, electrodes and blood pressure cuffs so that the mother’s chest and one arm are free. Prevent pain, nausea and low blood pressure so that the mother can feel comfortable in skin-to-skin contact with the newborn on her chest.
3. At birth, the newborn is immediately placed in skin-to-skin contact on the mother’s stomach/chest and the newborn’s tone and breathing are assessed according to routine. Wipe the newborn and also make sure that the mother’s skin is dry. Place the newborn in an upright prone and tucked position with the head to the side for an open airway. After caesarean section, the newborn is placed diagonally across the mother’s chest and, if possible, in a slightly elevated position with the head and upper body.
4. To prevent hypothermia, cover the newborn with dry textiles as soon as possible with the face free. For preterm and low birth-weight newborns and at low room temperature, heated textiles are used that are changed continuously and, if necessary, a cap is also used.
5. Umbilical cord clamping should be done without interrupting skin-to-skin contact. A longer umbilical stump may be cut initially so as not to break skin-to-skin contact.
6. Help the mother into a comfortable semi-sitting/reclined position so that the mother can see the newborn. Support with pillows and perhaps a bed rail to reduce the risk of the newborn slipping. After caesarean section, continuous monitoring of both the mother and the newborn is needed in terms of well-being, position, warmth and safety as long as the mother is cared for on the operating table.
7. Support parents to focus on the newborn and to pay attention to its instinctive newborn behaviors. Encourage skin-to-skin contact at least until the newborn has breastfed for the first time and/or fallen asleep. If the newborn has not breastfed, encourage early breast milk stimulation and expression to get milk production started. Colostrum is given directly into the newborn’s mouth.
8. Monitor the newborn’s position and make sure the airway is open. Inform parents about the importance of this and how to hold and position the newborn. For low birth-weight-, preterm-, and fragile newborns, binders are recommended for safe positioning. Pay extra attention if the condition of the newborn and/or the mother is affected by drugs or illness and do not leave them alone during the initial period. This is to avoid sudden unexpected postnatal collapse.
9. Postpone routine care of the newborn, such as measuring and weighing, until at least after the first two hours of uninterrupted skin-to-skin contact. Motivated procedures and interventions, medical checks and treatments such as respiratory support, vitamin K prophylaxis, transfers, blood sampling, feeding and more are done without interrupting skin-to-skin contact and with continued good temperature control. Many interventions and treatments of the mother can be performed with or without adaptations with the newborn in uninterrupted skin-to-skin contact. This includes bleeding control procedures for the mother. Continuous skin-to-skin contact is recommended beyond the first few hours.
10. Care interventions during the first hours that involve interruption in skin-to-skin contact should be justified by the grounds that the benefits of the intervention involving interruption outweigh the benefits of continued skin-to-skin contact.
11. Extensive care needs of the mother and/or the newborn may prevent immediate and uninterrupted skin-to-skin contact. In these cases, physical proximity between mother and newborn is desirable during stabilization and continued care.

### 9.2 Mother-newborn couplet care

Couplet care is provided according to the clinical condition and medical needs of the mother and the newborn, as well as according to local conditions and capacity of the unit. Couplet care of mothers and newborns can take place in all departments where newborns and new mothers are cared for, and the place for couplet care is not static. Couplet care is most often planned on the unit responsible for the patient with the most complex medical needs.

General recommendations for couplet care:

1. Clarify the division of responsibilities between different units. Regularly assess the health status and care needs of both mother and newborn. If different needs are pitted against each other, use cross-organizational dialogue to find common solutions.
2. Optimize a care environment for both mother and newborn that ensures safety, personal privacy, closeness and the opportunity to rest and sleep together. This can be achieved through, for example, an adult bed at the newborn’s place of care, binders, screens/curtains for privacy protection, dimmed lighting and adjusted acoustic environment. Provide parents with access to meals, toiletries, toilets and showers.
3. Ensure that emergency equipment, medications and emergency call procedures for both mothers and newborns are available and known.
4. Ensure that both the mother and the newborn are easily accessible to healthcare professionals and that both can be transferred quickly if necessary.
5. Post-operative care and monitoring can take place in the maternity or neonatal ward, provided there are agreed local guidelines.
6. Transfers within the hospital should take place without separation and, as far as possible, in skin-to-skin contact using mobile equipment.
7. Usual checks, monitoring, blood sampling, medications, breastfeeding support and more are carried out according to medical needs and where the mother and the newborn are cared for together.
8. Daily rounds should take place for both mothers and newborns in the unit where they are cared for in couplet care.
9. Binders that facilitate safe care in skin-to-skin contact should be offered and used, such as tube binders, special wraps or shirts. Continued skin-to-skin contact is recommended as continuously as possible until discharge and beyond.
10. When parents and newborns sleep unattended and unmonitored, the newborn should have its own place to sleep—close to the parent—to reduce the risk of sudden unexpected postnatal collapse.
11. When discharging the mother and/or the newborn: ensure that checks, follow-ups and planning are clear and communicated to the family.
12. In the case of palliative or end-of-life care for a mother or a newborn, high priority should be given to keeping them together and offering privacy as well as continuous support. Facilitate skin-to-skin contact to alleviate stress and pain.

## 10 Insurance medicine and certificates

Couplet care of the mother and the newborn is facilitated by the fact that the other parent or another person designated by the parent also stay with the newborn in the hospital. Information about parental leave and insurance and early issuance of a certificate for sick leave and care of a sick newborn can make it easier for the family, regardless of where the mother and the newborn are cared for in couplet care.

## 11 Considerations for the guideline

### 11.1 Risk-benefit balance for the patient

The overall assessment for the recommendations is that the benefits outweigh the risk to the mother and the newborn, regardless of mode of birth.

If couplet care of a mother and a newborn is not provided, there is a risk of repeated and in some cases prolonged separations. If couplet care is not provided, immediate and uninterrupted skin-to-skin contact during the first hours and continued skin-to-skin contact thereafter is not possible.

In case of incomplete implementation of immediate and uninterrupted skin-to-skin contact and couplet care, with insufficient adaptations and when requirements are not met, there is a risk of inadequate care and follow-up in both acute situations and routine care. For the mother, this means increased risk of bleeding, risk of urinary retention, inadequate treatment of conditions such as high blood pressure, pain and infections, as well as a lack of support for breastfeeding and psychological well-being. For the newborn, incomplete implementation can have consequences such as sudden unexpected postnatal collapse (7, 91), hypothermia, inadequate breathing and low blood sugar.

In this guideline, it is emphasized that in order for immediate and uninterrupted skin-to-skin contact and couplet care to be possible and safe, the specified requirements in organization, premises, equipment and competence need to be met. Likewise, local guidelines are needed for individualized assessment and tailoring of care to decide level of care and place of care for the mother-newborn dyad. Several Swedish hospitals have made guidelines specific for their local prerequisites (92).

For care to be safe for mothers and newborns evidence-based guidelines for obstetric and newborn care must be used according to the clinical conditions and medical needs of both patients. This includes following the recommendations for the prevention of sudden infant death syndrome when parents and newborns sleep unattended (93).

### 11.2 The cost-effectiveness of the measure(s)

Immediate and uninterrupted skin-to-skin contact has the potential to improve the quality of care with relatively small investments. Based on described evidence and national and international experience, health economic gains are expected through reduced morbidity for mothers and newborns with reduced need for specialized care, fewer interventions and potentially stressful procedures, as well as shorter hospital stays and long-term improved health. Through improved breastfeeding, skin-to-skin contact has a number of additional cost-effective outcomes for both mothers and newborns (25, 40, 94, 95).

Couplet care has the potential to lead to reduced healthcare costs through improved care quality and increased efficiency in care (25, 26). Financial savings can be achieved through, for example, reduced need for floor space when a mother and her newborn share the same room, fewer transfers between wards, and confident parents actively participating in the care of their newborn, enabling alternative use of staff resources. Effects of implementation are followed with indicators based on target group and change, see the Impact assessment for this guideline (27, 28).

New ways of working with immediate and uninterrupted skin-to-skin contact and couplet care could, based on experiences from other changes in accordance with an Infant- and Family-Centered Developmental Care approach, provide increased job satisfaction and thus continuity in staff groups (96).

### 11.3 Direct and indirect implementation costs

Experience from units that have been adapted to increasingly offer immediate and uninterrupted skin-to-skin contact and couplet care for mothers and newborns in need of specialised care suggests that many initial additional costs are temporary. Similarly, costs for adapting premises and equipment for the maternal care in a department other than the birth unit and maternity ward are temporary investments. Providing care with immediate and uninterrupted skin-to-skin contact for newborns in need of specialised care may require one-time investments for mobile equipment. In an early implementation phase, a certain increase in personnel costs has been described. Continuous training efforts may be needed for adequate cooperation between personnel in obstetric care, postpartum- and postnatal care, neonatal care, anesthesia, surgery and intensive care.

### 11.4 Equity

Despite clear consensus in national and international guidelines on the importance of immediate and uninterrupted skin-to-skin contact and couplet care, current healthcare processes and working methods still lead to deficiencies in practice (97–100). The UN Convention on the Rights of the Child describes the child’s right not to be separated from his or her parents and that the best interests of the child shall be a primary consideration (8). When the mother gives birth by caesarean section, or needs other surgical procedures or intensive care in connection with the birth, the mother and the newborn are often separated. Separations are also common as newborns require neonatal care immediately after birth, resulting in limited opportunities for immediate and uninterrupted skin-to-skin contact (101).

A survey in 2023 on couplet care in Swedish neonatal units showed that less than half of the units could offer couplet care for mothers with a newborn cared for in the neonatal unit and there was a large variation in the degree of implementation (17). It also emerged that the implementation of methods that enable immediate and uninterrupted skin-to-skin contact at birth for newborns in need of medical support had begun in many places but was still insufficiently implemented across the country.

### 11.5 Patients’ propensity to accept the interventions

The propensity of parents to accept the recommendations is high, because being with their newborn from birth is a natural thing. The natural place for a newborn is in skin-to-skin contact with the mother. Separation is partly caused by the way care is organized and partly by a lack of conditions to accommodate and support parents (97). Parents should be informed of the newborn’s rights not to be separated from them and their mutual need to be close to each other, regardless of the care needs of the mother and the newborn. Dialogue with parents about previous experiences, needs, expectations and physical and mental resources is important in order to provide the opportunity for informed choices and full participation in the care of a sick or preterm newborn. The family’s situation and opportunities to be together and care for their newborn skin-to-skin can be made easier by getting support and help in their situation from other family members. The family needs access to food, sanitation and an environment that enables privacy and sleep (21). There are international standards for how the care environment in the neonatal unit should be designed to accommodate this (102–105).

### 11.6 Feasibility

Experience shows that a stepwise implementation of immediate and uninterrupted skin-to-skin contact and couplet care is common (28). In existing operations, a first step may be to introduce couplet care for mothers and newborns who have a low risk assessment after birth. As experience, premises, equipment, competence and confidence develop, the care of increasingly complex patients can take place in immediate and uninterrupted skin-to-skin contact and in couplet care. The goal is for immediate and uninterrupted skin-to-skin contact and couplet care to be offered regardless of care needs. The care is planned and provided according to the clinical situation and medical needs of the mother and the newborn. Different models for how departments and units have been organized to be able to offer couplet care has been described (17, 31). Collaborations, organization of management and staff as well as how premises are planned are based on local conditions, capacity and the organization of care. Couplet care can be carried out in healthcare environments with different architecture, and single rooms are not a strict requirement. When new wards are built, the environment should be optimized for couplet care and for a pleasant atmosphere for newborns and parents as well as for staff. In-depth information on the design of adapted care environments can be found in European and American standards (106, 107).

Sustainable implementation of couplet care and working methods for immediate and uninterrupted skin-to-skin contact for all mothers and newborns is based on cross-functional collaboration between maternal health care, obstetric care, postpartum- and postnatal care, neonatal care, anesthesia, surgery and intensive care (29, 30).

### 11.7 For continued care after couplet care

When either the mother or the newborn is discharged, a form of care other than couplet care takes over because it is no longer two patients who are cared for together. If the mother needs continued care, she should be able to have the newborn close and breastfeeding should be supported as far as possible. Children’s right to be with their parents or with a person or family member designated by a parent continues to apply at all times during any hospital stay.

The other parent stays at the hospital as a partner to the mother and parent to the newborn when the mother and the newborn are cared for together. Parents of hospitalized newborns and children of all ages should be offered to live with the child in a family room (1). More information on the principles of Infant- and Family-Centered Developmental Care can be found in the European standards of care for newborn health (108).

## 12 Impact assessment

### 12.1 Expected changes

The guideline and implementation of its recommendations are expected to provide:

- positive health effects for mothers, newborns and families
- equality of care regardless of when, where and how birth takes place and what the subsequent care requires
- better conditions for fulfilling Article 9 of the Convention on the Rights of the Child on the child’s right not to be separated from their parents and Article 3 on that the best interests of the child shall be a primary consideration
- increased knowledge and awareness among staff at all levels in the care chain
- new ways of working with increased teamwork across operational boundaries and disciplines
- support for business development across maternal-newborn health care facilities, quality follow-up and collaboration between care providers
- indicators for local, regional and national governance and follow-up.

### 12.2 Public health, care needs, and healthcare access

Through couplet care, the mother and the newborn can receive coordinated care based on both their individual needs and situation, which contributes to improved quality and safety. Person-centeredness increases when care is conducted in a cohesive manner and the family is given the opportunity to actively participate in the planning of their own care. Couplet care provides good conditions for immediate skin-to-skin contact at birth and uninterrupted skin-to-skin contact thereafter.

Maternal-newborn skin-to-skin contact has been shown to have positive health effects by:

- enhanced breast milk production and breastfeeding
- colonization of the newborn with a beneficial bacterial flora
- improved resistance to infections in the newborn and early childhood
- improved stabilization of breathing, circulation and temperature during the physiological adaptation in the newborn after birth
- improved neurological and cognitive development of the child
- improved parent-child interaction
- improved maternal blood pressure regulation
- reduced amount of bleeding after childbirth
- reduced pain experience for the mother and the newborn
- reduced stress for the mother and the newborn.

### 12.3 Health economic perspectives

Health economics focuses on using healthcare resources so that the greatest possible health effect is achieved for the population. The following financial effects and operational consequences are based on studies, quality registers and reported experiences from units that implemented couplet care and/or immediate and uninterrupted skin-to-skin contact. Assessment of potential economic improvements from benefits and impacts of immediate and uninterrupted skin-to-skin contact and couplet care must be based on the current level of implementation for each operation and unit. The change can be evaluated over time with the support of indicators of care data from relevant quality registers.

Health effects and operational consequences with expected cost reductions are:

- more cohesive care and improved patient safety through coordinated reports between care teams, shared care location, reduced number of transfers between units and improved sense of security and continuity for patients
- fewer interventions and procedures during the hospital stay for both mother and newborn
- fewer newborns needing specialised care
- shorter length of stay for newborns in hospital
- reduced use of milk formula through improved breastfeeding and lactation.

In the longer perspective and at the societal level, costs for healthcare and for sick leave can be reduced by:

- fewer infection-related healthcare contacts for the child
- improved neurological and cognitive development of the child
- improved mental health for the mother and the other parent.

### 12.4 Ethical perspective

This guideline emphasizes that hospital services and staff are responsible for enabling skin-to-skin contact and couplet care as far as possible. Support must be given on the basis of a person-centered approach, taking into account the patient’s needs and resources. A clear goal is that organization, equipment and staffing should not constitute an obstacle to immediate and uninterrupted skin-to-skin contact and couplet care.

This guideline’s recommendations on couplet care strengthens the patient’s right to information, participation and respect and, the family's possibility to be together. Implementation of couplet care also means that newborns are only separated from their parents if it is beneficial to the newborn and that reunification is facilitated as soon as possible. Couplet care helps to ensure maternal-newborn skin-to-skin contact early, repeatedly and even continuously.

Early, accessible and tailored information about benefits for both the mother and the newborn, is needed from healthcare providers to facilitate immediate and uninterrupted skin-to-skin contact. Every health care situation must protect the privacy and integrity of the patient, and special consideration is warranted when medical procedures require exposure of skin and intimate parts of the body. In situations where immediate and uninterrupted skin-to-skin contact between mother and newborn is not possible due to personal, physical or psychological circumstances, a supportive, non-judgmental approach is particularly important. Individual medical risk assessments of both mother and newborn make it easier for staff to tailor care and find cross-organizational common solutions for skin-to-skin contact and couplet care. In complex situations, involvement of the other parent and the family support network can enable the newborn to have skin-to-skin contact with another family member.

### 12.5 Consequences in individual healthcare services

This guideline aims to:

- increase knowledge and awareness of the effects of immediate and uninterrupted skin-to-skin contact and couplet care
- spread practical knowledge about how immediate and uninterrupted skin-to-skin contact and couplet care can be performed
- facilitate implementation and increase collaboration between healthcare operations
- emphasize that conditions for immediate and uninterrupted skin-to-skin contact and couplet care need to be prioritized in operational and organizational changes.

Currently, there are limited opportunities to map how well the working methods with immediate and uninterrupted skin-to-skin contact and couplet care are applied. Different definitions of couplet care and few parameters for immediate and uninterrupted skin-to-skin contact make systematic follow-up difficult. The guideline’s clarified definitions and proposed indicators aim for quality registers to be used for monitoring implementation and compliance, both locally and nationally.

During an implementation phase, new ways of working can pose challenges when staff need to perform regular tasks in an environment that is unfamiliar to them. This can be facilitated through training, including structured simulation exercises and auscultation visits to other units, as well as adaptation of equipment and premises to maintain good ergonomics and working environment. Common cross-operational procedures and clear communication channels need to be established. Ways of working with mobile teams and equipment and in some cases delegation of tasks and responsibilities mean that staff need guidance and forums for follow-up and evaluation.

Implications for individual health care providers of implementing the recommendations may include:

- initial costs of mobile equipment
- initial costs för reconstruction of premises
- reduced costs for medical devices, particularly incubators and CPAP accessories
- increased costs and efforts for improved competence within, and coordination between, care teams
- reduced costs and efforts for staffing through increased job satisfaction and continuity for staff.

Over time and with progressively improved implementation, the costs for equipment and adaptation of premises as well as for coordination and collaboration between teams should decrease. The application of the recommendations in this guideline does not need to involve any organizational changes beyond a strengthened collaboration between the hospital departments that care for mothers and newborns.

For successful implementation, the changes in working methods need to be well anchored at the overall management level and take place across operations, with a clear division of responsibilities and in collaboration with all relevant personnel. By involving patient representatives, additional perspectives and valuable information can be added. National, regional and local networks and groups work to facilitate exchange of experience and collaborations and can be used as a resource in the design of checklists and action plans.

For operations in an early stage with a low level of implementation, a risk and impact analysis, in which all units participate can identify gaps, improvement measures and costs. One option suggested in this guideline and which has been used by several departments is to implement changes in smaller steps, starting with less fragile newborns and mothers.

## 13 Follow-up

This guideline addresses staff and managers in the care chain of maternal health care, obstetric care, postpartum- and postnatal care, neonatal care, anesthesia, surgery and intensive care and includes the two patient groups: mothers and newborns. Follow-up on implementation and quality of care should include all operational and patient perspectives. National and/or local registries following quality of maternal and newborn care are recommended to include indicators to specifically monitor implementation of skin-to-skin contact and mother-newborn couplet care.

### 13.1 Quality monitoring and indicators

Quality monitoring of immediate and uninterrupted skin-to-skin contact and mother-newborn couplet care should include both process- and outcome measures. Relevant indicators include initiation and duration of skin-to-skin contact, breastfeeding or breast milk by mouth within the first hour, breastfeeding outcomes, extent of couplet care and the occurrence of separations, and patient-reported experiences.

To enable consistent follow-up across care settings, data collection should capture key elements such as timing of initiation and interruption of skin-to-skin contact, reasons for interruption or absence of skin-to-skin contact, early feeding practices, whether the mother and the newborn were cared for together throughout hospitalization, and factors associated with incomplete couplet care. Parent’s well-being and individual experiences should also be included.

Relevant national registries could record the following indicators:

- initiation time for skin-to-skin contact with mother
- initiation time for skin-to-skin contact with the other parent or the person designated by the parent
- time of first interruption of skin-to-skin contact
- cause of interrupted or absent skin-to-skin contact during the first two hours after birth
- whether or not the newborn breastfed or received the mother’s breast milk by mouth during the first hour after birth
- if the mother and the newborn were cared for together from birth until the mother or the newborn was discharged
- separations between a newborn and the mother
- causes of incomplete practice of couplet care
- breastfeeding outcomes
- the mother’s experiences of separations
- the mother’s experiences of non-separation in couplet care
- mental health in mothers during the first year following birth
- well-being in both parents during the first year following birth.

## 14 Swedish quality registries and indicators

Several quality registries are used in the Swedish care chain for mother and infant during pregnancy, childbirth, post-partum and neonatal care. Many existing indicators can be used to monitor implementation of immediate and uninterrupted skin-to-skin contact and mother-newborn couplet care.

The Pregnancy register:

The pregnancy questionnaire sent to all new mothers eight weeks after birth asks whether the newborn was in skin-to-skin with the mother immediately after birth and until the newborn had suckled and/or fallen asleep.

The Swedish Neonatal Quality Register:

- amount of skin-to-skin contact during the first day
- recordings of skin-to-skin contact amounting to more than 3 hours per day
- recordings of skin-to-skin contact amounting to more than 6 hours per day
- timing of the first mother’s milk by mouth.

The Child Health Care Quality Register:

- breastfeeding and other methods of feeding since birth and onwards
- depressive symptoms in the mother during the first year after birth

The Pregnancy Register and the Swedish Neonatal Quality Register also include patient questionnaires that enable registration of patient-reported experience measures and patient-reported outcome measures. The Swedish Neonatal Quality Register’s annual report contains a description of the capacity and structure of the neonatal care units, including equipment, staffing and competence based on data obtained through a survey to operational managers.

## 15 Discussion

The place for newborns—whether born at full term or prematurely—is in the mother’s arms, without the baby being separated from the parents. Over the past two or three decades, neonatal care has begun to rectify mistakes made a century ago; while survival rates in neonatal care improved, the experiences biologically essential for both infant, mother and family were simultaneously overlooked. As always, changes in clinical practice lag behind scientific knowledge regarding what constitutes best care.

An important core message in this guideline is that skin-to-skin contact and the concept to care for mothers and newborns together is all about the place of care. The content of care is always governed by the medical condition of each patient according to care protocols. In most cases, this does not contradict the recommendations in this guideline. Complex medical situations, especially if unexpected, can challenge other good intentions. There will be imperative situations where lifesaving interventions are prioritized. If separation is needed the best place of care with skin-to-skin contact or direct closeness between the newborn and the parent should be reinstalled as soon as possible. This is why we propose that teams always discuss the questions under “level of care and collaboration”.

Sudden Unexpected Postnatal Collapse (SUPC) can occur during early skin-to-skin contact as well as during separation (93). Continuous clinical monitoring and support of the newborn’s airway and positioning is essential, especially for fragile newborns. Ensuring the infant’s face remains visible and the nose is unobstructed is critical to preventing respiratory compromise while maintaining this vital experience for the newborn and the parent. According to our experience, the most evident risk for mother or newborn is if there is an incomplete implementation of immediate skin-to-skin contact and mother-newborn couplet care were care needs might be neglected or compromised related to lack of proper organization of care or quality of care. We believe that safe care can be provided following this guideline and its recommendations for requirements and for implementation.

The original Swedish guideline on immediate and uninterrupted skin-to-skin contact and mother-newborn couplet care was published almost two years ago. It was well received and has been reported to facilitate implementation and collaboration across units and disciplines in Swedish hospitals. We believe this was attributed to several factors: the author group had a broad and relevant representation; an extensive review process proceeded the publication; the launch webinar with open discussions; as well as a timing where several hospitals were ready for the next step in the process towards zero separation between mothers and newborns. As part of the preparations for the guideline a survey showed that in 2023, approximately one third of the Swedish neonatal units had implemented the first steps of couplet care in the neonatal unit. A follow-up on the implementation is planned in a near future. To our knowledge, many more units have made well-structured implementations following this guideline.

To change routines and mind-set in healthcare is difficult. There are no “one model fits all” and different settings have their unique opportunities and challenges. Implementation in a step-by-step strategy for different groups of patients and different conditions have been a successful way to go and teams in different parts of the world continue to publish their specific experiences. We believe that most if not all teams can and should increase collaboration across maternal and newborn care and make changes to facilitate the implementation to care for all newborns and mothers together and in skin-to-skin contact.

There are some evident changes following the publication of the Swedish version of the guideline on immediate skin-to-skin contact and mother-newborn couplet care. In Sweden, we now have consensus on clear definitions of the concepts. Awareness on benefits on zero separation and needed changes for best care practice increased among decision makers, managers and healthcare professionals in the care continuum for mothers and newborns as well as in the general public following this guideline. We now have a common vision to care for all mothers and newborns together, with as little separation as possible. We strongly recommend others to write national as well as regional and local guidelines and protocols for the clinical practice of immediate and uninterrupted skin-to-skin contact and mother-newborn couplet care for zero separation and the best possible well-being and outcomes for newborns, mothers and families.

## Data Availability

The original contributions presented in the study are included in the article/Supplementary Material, further inquiries can be directed to the corresponding authors.

## Supplementary material

Supplementary File 1 Document of approval by the Swedish Association of Local Authorities and Regions and the National Program Area for Children's and Adolescent's Health.
