# Supplementary material for: The Swedish national guideline on immediate and uninterrupted skin-to-skin contact and mother-newborn couplet care
Source: Front Pediatr. 2026 Sep 10;14:1937937. doi: 10.3389/fped.2026.1937937 (PMC13632495; doi:10.3389/fped.2026.1937937)
Supplement: Supplementary file 1 [file supplementaryfile1.pdf]

Permission to reprint translation of published material

Title of original work

Initial obruten hud-mot-hudkontakt och samvård
Published on 1177 Vårdguiden [vardpersonal.1177.se]

Publisher Approval Statement

On behalf of the Swedish Association of Local Authorities and Regions (Sveriges kommuner och regioner) and the National program area for Children's and Adolescent's Health (Nationellt programområde Barns och ungas hälsa), permission is hereby granted for:

- Translation and adaptation of the above-mentioned guideline
- Submission to and publication in *Frontiers in Pediatrics*
- Dissemination of the translated material as part of a peer-reviewed scientific publication

This approval confirms that the Swedish Association of Local Authorities and Regions, as the original publisher and rights holder, consents to the use of the material for the purposes described above.

Authorization

This permission is granted by an authorized representative of the National program area Children's and Adolescent's Health and the Swedish Association of Local Authorities and Regions.

Signature: ____________________

Katarina Hanséus
MD, PhD, Chair of the National program area Children's and Adolescent's Health, the Swedish Association of Local Authorities and Regions

Date: July 1st, 2026
